# Management of Pituitary Adenomas: Mononostril Endoscopic Transsphenoidal Surgery

**DOI:** 10.29252/nirp.bcn.9.2.121

**Published:** 2018

**Authors:** Houssein Darwish, Usamah El-Hadi, Georges Haddad, Marwan Najjar

**Affiliations:** 1. Department of Neurosurgery, School of Medicine, University of Virginia System, Charlottesville, USA.; 2. Department of Neurosurgery, Bahman Hospital, Tehran, Iran.; 3. Department of Otolaryngology- Head & Neck Surgery, Faculty of Medicine, American University of Beirut, Beirut, Lebanon.; 4. Department of Surgery, Division of Neurosurgery, Faculty of Medicine, American University of Beirut, Beirut, Lebanon.

**Keywords:** Endoscopic transsphenoidal surgery, Pituitary tumors, Mononostril approach, Binostril approach

## Abstract

**Introduction:**

The endoscopic transsphenoidal approach for pituitary adenomas and other sellar lesions is quickly becoming the procedure of choice in their surgical management. The most common approach is binostril three-hand technique which requires a large exposure and subjects both nasal cavities to potential trauma. To reduce nasal morbidity, we employ a mononostril two-hand technique with the help of the endoscope holder. In this research, we review our series to determine efficacy of this approach in the management of pituitary adenomas.

**Methods:**

We performed a retrospective analysis of our initial series of 64 consecutive patients with pituitary adenomas operated by the same surgical team from 2008 till 2014 using a mononostril endoscopic approach. After categorizing the lesions into microadenomas, noninvasive macroadenomas, and invasive macroadenomas, we reviewed the radiological and biochemical outcomes of the surgeries after 3 months, 12 months, and 18 months. We also assessed recurrences and complications. Extent of resection was divided into gross total resection, near total resection (>90% resection), and partial resection for the remaining.

**Results:**

Our results show resection rates comparable to most series in the literature, with a gross total resection of 87% in non-invasive macroadenomas, and surgical disease control in 75% of invasive nonfunctioning adenomas. The remission rate in Cushing’s disease was 81%, where it achieved up to 58% surgical remission in growth hormone secreting pituitary adenomas (including the invasive adenomas). The complication rate was very low.

**Conclusion:**

We conclude that the mononostril endoscopic approach is well suited for most pituitary tumor operations and carries comparable remission and resection rates to most endoscopic series with minimal complications and nasal morbidity.

## 1. Introduction

In 1978, Bushe and Halves introduced the use of the endoscope in pituitary operation (Bushe & Halves, 1978). However, it was not until the mid-1990s that the endoscope gained popularity for pituitary operation after otolaryngologists started using it for sinus operation with improved visualization and good working space. Yaniv and Rappaport described a combined approach in which the endoscope was used for the initial approach to the sphenoid sinus, followed by conversion to the standard transsphenoidal microsurgical approach for the tumor resection (Yaniv & Rappaport, 1997). Jho and Carrau later reported the largest series of patients who had undergone pure endoscopic endonasal transsphenoidal operation (Jho & Carrau, 1996).

Since the introduction of the endoscopic transsphenoidal surgery, most surgeons advocate either of two techniques; two surgeons (3 or 4 hands) technique, or one surgeon (2 hands) technique utilizing an endoscope holder (Cappabianca et al., 2002; de Devitiis, Cappabianca, & Cavallo, 2002; Yano et al., 2009). In the binostril 3-hand technique, the ENT surgeon does the exposure then holds the endoscope in one nostril (usually the right) and the neurosurgeon works with instruments using both nostrils. Usually the neurosurgeon holds the suction in the non-dominant hand and a dissecting instrument in the dominant one. With the mononostril 2-hand technique, the ENT surgeon may perform the nasal phase of the surgery, but then the endoscope holder (hydraulic or mechanical) may be used for the rest of the operation (Cappabianca et al., 2002).

Furthermore, as discussed by Edward Laws and John Jane Jr, the main advantage of the mononostril technique is less trauma to the nasal mucosa and thus less nasal morbidity such as crusting, loss of smell, and synechia (Jgannathan, Laws, & Jane, 2012). However, the main disadvantage is small working room, especially for invasive and large macroadenomas. In this situation, the degree of freedom that is gained by the operating surgeon is crucial for better surgical outcomes. This freedom is gained by elimination of the endoscope shaft, usually used for irrigation and holding, from one of the surgeons operating corridors. Based on this, we wanted to assess our results looking for the efficacy of the mononostril technique and compare it to the literature with respect to outcome and efficacy.

In this paper, we present a retrospective review of our series of sellar/suprasellar pituitary adenomas operated via the endoscopic endonasal transsphenoidal approach using a single nostril, 2-hand technique using the endoscope holder. We studied the short- and long-term outcomes, besides the complications of our operations. We also review the other surgical endoscopic approaches; the mononostril versus binostril techniques.

## 2. Materials and Methods

After obtaining IRB approval from American University of Beirut Medical Center (AUBMC), we performed a retrospective case review of all the pituitary adenoma operations done at AUBMC by the same surgical team from April 2008 till 2014, with no exclusions. We classified the tumors as microadenomas, non-invasive macroadenomas (Knosp Grades 0, 1), and invasive macroadenomas (Knosp grades 2 and greater). Outcomes were studied both radiologically and biochemically at 3 months, 12 months, and 18 months after the operations. Remission rates of patients with Cushing disease and Growth Hormone (GH) secreting adenoma were also determined.

For patients with Cushing’s disease, we used the early morning cortisol level less than or equal to 2 μg/dL as cut-off point for early onset remission (Biller et al., 2008). For GH secreting tumors, we used the random GH, but not within 3 months of surgery, level of less than 1 ng/mL as cut-off point for remission (Giustina et al., 2010). Resection on Magnetic Resonance Imaging (MRI) was classified as Gross Total Resection (GTR), near total resection with residue less than 10% whether inside or outside the cavernous sinus and partial re-section for the remaining, based on 3-month pituitary protocol MRI. We then performed a literature review in PubMed, Cochrane and Google using pituitary adenoma surgery, Cushing’s disease, Growth hormone secreting adenoma, endoscopic endonasal surgery, 3-hand approach, 2-hand approach, mononostril, and binostril pituitary surgery as keywords.

### 2.1. Surgical technique

**Figure 1.**
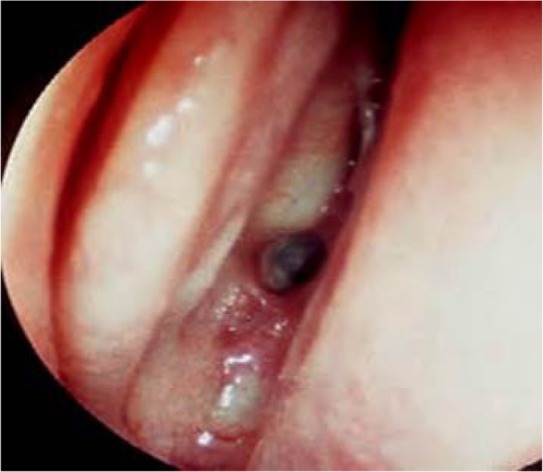
Endoscopic view through the right nostril showing the nasal septum on the right, the lateralized middle turbinate on the left, and the sphenoid ostium just underneath the superior turbinate in the middle

**Figure 2.**
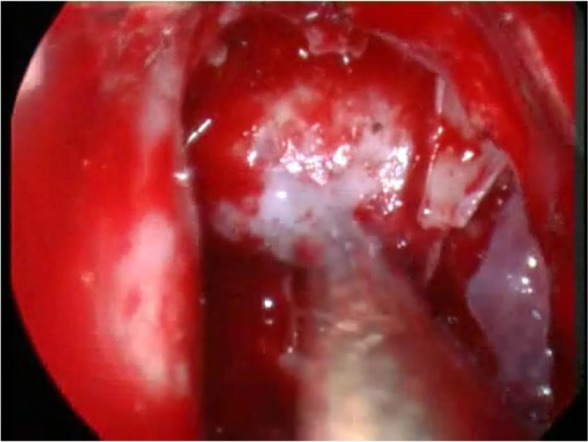
After removing the bone between the sphenoid ostia, a wide endoscopic exposure of the sella turcica is attained

We used the mononostril approach in all operations. The choice of the nostril was done with the help of a preoperative 1-mm cut CT scan of the sinuses. If there was no septal deviation we tended to use the right nostril, as both surgeons were right handed, and it was easier to handle the scope with the left hand and work with the right. Should there be a septal deviation, a Submucosal Resection of the septum (SMR) or operation through the other nostril may be performed. The middle turbinate is lateralized, and as we reach and identify the sphenoid ostium on one side, we continue in a submucosal fashion across the perpendicular plate of the ethmoid to expose the contralateral ostium (Figure 1). Removing the bone between the two ostia and connecting them in one big hole creates a wider working space (Figure 2). The mucosa over the entered ostium is either coagulated or used to make a flap for better closure, whereas the mucosa on the other side is kept intact for better healing of the nose. As we enter the sphenoid sinus the scope is held with the scope holder and pushed to the upper corner of the nostril. Under such circumstances, we can use 2-hand technique with suction in the left hand and the other working instrument in the right one. After removal of the adenoma in the usual method, surgicel was applied and then fixed with glue. A fat graft, taken from a small abdominal incision, was tucked in and fixed with glue, should an intra-operative Cerebrospinal Fluid (CSF) leak occur.

## 3. Results

We had 64 consecutive patients in our series: 14 with microadenomas (12 adrenocorticotrophic hormone [ACTH] secreting, 2 GH secreting), and 50 with macroadenomas (23 non-invasive, and 27 invasive).

### 3.1. Microadenomas

We had performed 16 operation on 14 patients with microadenomas; 11 females, and 3 males, with a mean age of 33 years. There were 12 ACTH secreting microadenomas; 10 females and 1 male, whereas there were 2 GH secreting microadenomas, both of whom were males. The mean follow up period was 17 months (range:4–72 months).

**Figure 3.**
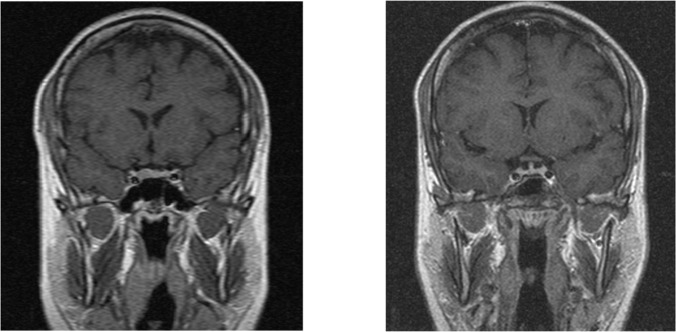
a) Coronal T1W enhanced MRI sequence through the sella turcica showing a right sellar hypoenhancing nodule suggestive of pituitary microadenoma in a patient with Cushing’s disease, b) Post-operative MRI showing no residual adenoma The patient had biochemical remission as well.

Among the 12 ACTH secreting microadenomas, 9 had biochemical remission postoperatively and at last follow up after the first operation (75%), whereas there were 3 failures (25%) (Figures 3a & 3b). Of the latter cases, 1 patient had 3 operations; the first successful operation was followed by recurrence after one year, then a failed second surgery; afterwards the third operation achieved biochemical remission till the last follow up, raising the long-term remission rate to 83% (10/12 patients). Two other patients had previous failed operations at other centers, and despite lateralization by IPSS, our operations failed in achieving surgical cure.

There were two patients with GH secreting adenomas. The first patient achieved post-operative radiological and biochemical remission. The other patient, who had 2 previous operations at another center and was on medical treatment, failed to achieve cure and was kept on somatostatin treatment. Overall, long-term remission was achieved in 11/14 patients with secretory microadenomas (79%). It was noted that all patients who did not achieve remission were those who had previous failed operations.

### 3.2. Non-invasive macroadenomas (Knosp grades 0, 1)

We had 23 patients with non-invasive macroadenomas; 9 non-secreting, 7 GH secreting, 4 prolactinomas, and 3 ACTH secreting adenomas. These were distributed among 14 males and 9 females, with a mean age of 44 years. The mean follow up was 19 months (ranging between 3 and 72 months).

Gross total removal, as evidenced by 3 months postoperative MRI and at their last follow up, was achieved in 7/9 non-secreting macroadenomas (78%); 2 had a small stable non-growing residual (near total removal) on post-operative and at their last follow up MRI (22%). All 7 patients with GH secreting tumors achieved gross total removal by MRI (100%). Biochemical remission, however, was achieved in 6/7 (86%), with elevated IGF-1 in one patient who was kept on medical treatment (14%). As for prolactinomas operated for hemorrhage or resistance to treatment, 3/4 achieved remission (75%), whereas one patient had recurrence and was kept on medical treatment. All three ACTH secreting non-invasive macroadenomas achieved biochemical cure after surgery and at last follow up (100%). Overall, gross total removal was achieved in 87% of non-invasive macroadenomas (20/23), whereas the rest had near total removal. Biochemical remission was achieved in 85% of secretory non-invasive macroadenomas.

### 3.3. Invasive Macroadenomas (Knosp grades 2≤)

These included 27 patients, 19 males and 8 females. Sixteen patients had non-secreting adenomas, and 11 secreting adenomas (3 GH, 7 prolactin, and 1 ACTH secreting tumors). The mean follow up period was 22 months, ranging from 3 to 66 months. As for the patients with non-secreting invasive macroadenomas, 14 out of the 16 operations were near total resection with suspicious minor or cavernous sinus residual on 3 months post-operative MRI (87%). Of these, however, 3/14 patients (21%) had growth of the residual treated with radiation therapy, whereas 11/16 were stable at last follow up (61%). Two other patients had debulking with a significant residual on post-operative MRI, necessitating redo surgery in both, where one had stable residual and the other needed radiation therapy. Thus, surgical long-term stability with minor residual was achieved in 12/16 patients (one patient had 2 surgeries), with radiation therapy necessary in 4 patients (25%).

As for the secreting adenomas, 3/11 had GH secreting adenomas. Two patients had major resection (near total) with cavernous sinus residual on post-operative MRI, and since biochemical remission was not obviously achieved despite the drop in GH and IGF-1 levels, they were maintained on medical therapy. The third patient, who had an extensive tumor, had debulking surgery necessitating another surgery and then stereotactic radiosurgery and medical treatment for the eventual small residual of the invasive tumor. The seven invasive prolactinoma cases were maintained on post-operative medical therapy, after a near total but non-curative re-section, aimed at debulking of the necrotic/hemorrhagic lesion to relieve mass effect and improve response to treatment. Three patients had cavernous sinus residual only (near total removal), whereas four had cavernous sinus and minor suprasellar region residual. The ACTH secreting adenoma patient had preoperative clivus invasion, and had no gross residual on postoperative MRI, other than the suspicious invaded clival area. The patient underwent fractionated radiation therapy with drop in cortisol, maintained at the last follow up.

### 3.4. Hospital stay and operative time

Hospital stay ranged between 2 and 5 days with an average of 2.8 days. The operative time ranged between 2.2 to 4 hours with an average of 2.8 hours.

### 3.5. Complications

Complications were mostly transient, including epistaxis in one patient, crusting in 6 patients, and transient hypopituitarism in another. Around one third of the patients (24/68), especially those with macroadenomas, had transient diabetes insipidus that lasted few days on average. One patient who had redo surgery after previous operations had permanent diabetes insipidus. Meningitis occurred in one patient who had a second redo surgery, and had been irradiated in the past. Although a few CSF leaks (8/68) were noted at surgery and repaired intra-operatively using a small fat graft and glue, only one CSF leak occurred after the operation (which was a redo surgery). This was treated in a similar fashion and augmented by lumbar drain for 5 days.

## 4. Discussion

Our results are comparable to most endoscopic and microscopic series reported in the literature. We have achieved a gross total resection of 87% for non-invasive macroadenomas overall, with a stable residual in 13%. Among those, the nonsecreting adenomas had a 78% gross total resection rate, with 22% having a small stable residual at their last follow up (near total removal). The invasive nonsecreting adenomas on the other hand had an initial 87% near total resection (14/16 patients), where 3 patients, in addition to the two patients who had partial removal (31%), had relapse and underwent redo surgery, radiation therapy, or both to achieve stability. Long-term surgical stability at last follow up was thus achieved in 12/16 patients with invasive nonfunctioning adneomas (75%), whereas radiation was needed in 25%. Of note is that most of the residuals or recurrences requiring another operation or radiation therapy occurred in the invasive adenoma group (Table 1).

**Table 1.** Surgical remission and extent of resection in non-secreting adenomas

	**Non-invasive Macroadenomas**	**Invasive Macroadenomas**
Gross total removal	78%	None
Near total removal	22%	87%
Surgical remission	100%	75%
Progression/radiation	None	25%

Biochemical remission was achieved in 6/7 of GH secreting non-invasive macroadenomas (86%), and 7/12 (58%) of GH adenoma patients overall (Table 2). The factors which were against biochemical remission were invasive tumors, and previous operation (for microadenomas). Even in the patients who did not achieve surgical remission (mostly invasive adenomas), operation was an important part of the multifaceted treatment, aiming at reducing residual invasive tumor size to a minimum, to allow highest success of adjuvant therapies. Biochemical remission was achieved in 13/16 (81%) of ACTH secreting adenomas overall (one patient had 3 operations to achieve remission). History of previous operation and invasiveness were again factors affecting negatively biochemical remission. As for prolactinomas, the major indication for surgery was symptomatic apoplexy, or symptomatic macroadenomas not responding to medical treatment, and goal of surgery thus was mostly lesion debulking, which was achieved in 7/10 patients. The other three patients had total resection.

**Table 2.** Surgical biochemical remission rates in GH and ACTH secreting adenomas

	**Microadenomas**	**Non-Invasive Macro (%)**	**Invasive Macro**	**Total (%)**
GH	50%	86	None	58
ACTH	83%	100	None	81

### 4.1. Endoscopic surgery outcomes (literature and our series)

Pituitary adenoma operation can be performed either microscopically through a sublabial approach or endoscopically. Endoscopic approach can be mononostril (two-hand technique), binostril (two-hand technique) with the use of endoscope holder, or binostril (three-hand technique) (Mamelak, Carmichael, Bonert, Cooper, & Melmed, 2012). Endoscopes not only allow a panoramic view, but also allow the advancement of this view into the surgical field.

The use of 30 degrees and 45 degrees scopes also allows the surgeon to look inside the sella and more importantly allows access to hidden tumor in the lateral aspects of the sella, thus attaining a safer and more complete resection of tumor under direct vision (Cappabianca, Cavallo, & de Divitiis, 2004). This has been demonstrated in our series in the high rate of gross total resection achieved in the non-invasive macroadenoma group, where the endoscope allows further inspection of the hidden corners and the suprasellar region. Furthermore, less invasive endoscopic approach has the potential to shorten operation time, obviates the need for nasal packing, and shortens hospital stay (Koren, Hadar, Rappaport, & Yaniv 1999). In a comprehensive meta-analysis in 2006, Tabaee et al. demonstrated both safety and efficacy of the endoscopic approach with high rates of gross total removal, normalization of endocrine function, and improved vision (Tabaee et al., 2009).

As for endocrinological outcome, biochemical cure in Growth Hormone (GH) secreting adenomas ranges around 40%–75% depending on suprasellar extension and invasion of the cavernous sinus, often seen in this kind of adenoma (Hofstetter et al., 2011). Our high remission rate achieved in non-invasive GH secreting macroadenomas (86%) was expectedly lowered to 58% for the whole group when invasive adenomas were included. The initial cure rate in Cushing’s disease ranges around 65%–90%, and in one series is lower for microadenomas (55%) versus macroadenomas (71%) owing to a high rate of lesions that may be overlooked on preoperative MRI imaging (Biller et al., 2008, Hofstetter et al., 2011). We have also noted this in our series where we had remission in 75% of microadenomas versus 100% of non-invasive macroadenomas presenting with Cushing’s disease. Previous surgery and microadenoma were factors in lowering remission rates. In prolactinomas, the cure rate hovers around 50% for macroadenomas when they are commonly invasive and hemorrhagic, with marked suprasellar extension (Hofstetter et al., 2011). The main goal of surgery, indicated mostly for symptomatic apoplexy, remains tumor debulking to save visual function and reduce tumor load as seen in our series (achieved in 8/11 patients, where the other 3 patients had total re-section). Thus, for secretory adenomas in general, and even in the patients who did not achieve surgical remission (invasive tumors), operation was an important part of the multifaceted treatment, aiming at reducing residual invasive tumor size to a minimum and allow the highest success for adjuvant therapies.

As for patients with preoperative visual field disturbances, complete recovery of vision was seen in 40%–50% of the cases and improvement in 39%–51% of the cases in two large endoscopic series (Dehdashti, Ganna, Karabatsou, & Gentili 2008; Mortini, Losa, Barzaghi, Boari, & Giovanelli 2005). In our series, we had 60% complete recovery of vision and improvement was seen in another 30% of patients. One patient who had presented with third nerve palsy had near total recovery after operation. One common complication is transient diabetes insipidus. Permanent diabetes insipidus is much less common and is seen in around 1% of the cases (Tabaee et al., 2009). Postoperative CSF leak rate ranges around 2%–4%, and in the 200 patients reported by Dehdashti et al., it was 3.5% (Dehdashti et al., 2008, Tabaee et al., 2009). Only 2% of our patients had persistent diabetes insipidus and one had postoperative CSF leak.

Hospital stay is relatively short in most endoscopic series, and in one retrospective study, Neal et al. showed a significant decrease in hospital stay (3.4 days) and operation time (4.41 hours) using the endoscopic approach (Neal, Patel, Kulbersh, Osguthorpe, & Schlosser, 2007). In our series, the hospital stay ranged from 2 to 5 days with an average of 2.8 days. The operative time ranged around 2.2–4 hours with an average of 2.8 hours.

### 4.2. Mono-Versus Bi-nostril Endoscopic Technique

As mentioned earlier, the endoscopic transnasal approach offers excellent results when it comes to removal of pituitary tumors, with less nasal complication rates when compared to the microscopic sublabial transphenoidal approach (Koren et al., 1999). However, there is controversy as to whether the binostril or mononostril endoscopic approach is superior. Some neurosurgeons prefer the mononostril approach, whereas the otolaryngologists prefer the binostril approach (Cappabianca et al., 2004; Nakagawa, Takashima, Tomiyama, & Asada 2001). Far from being a rule, however, this has created controversy over the preferred endoscopic approach for pituitary lesions.

As for the binostril approach, the ostia are separately and bilaterally identified and the mucosa can then be coagulated or turned as a flap. The scope is held, usually by the ENT (Ear, Nose, & Throa) surgeon or by scope holder, in one nostril usually the right and the neurosurgeon works through both nostrils with his two hands. The major advantage in this approach is the dynamic process achieved with both surgeons working together at the same time. The space afforded for surgical instruments is also wider, with easier maneuvering. The major disadvantage is mucosa disruption on both sides of the sphenoid ostium, which may lead to more nasal crusting and discomfort.

We have described earlier our usual single nostril approach, where after lateralization of the middle turbinate, localization of one sphenoid ostium, and exposure of the ethmoid plate, the contralateral ostium is performed. The central bone removal affords an acceptable wide working space. It is generally felt that preservation of the contralateral mucosa is important for proper healing of the nose. As pituitary tumors are usually soft and easily removed with pituitary curettes and suction, the authors believe that the space provided by the single nostril approach is enough, though sometimes a bit crowded, to perform the procedure with high success rate. The authors, further, remove the endoscope holder towards the end of tumor resection, and inspect the surgical field in a dynamic fashion through the same nostril allowing further removal of possible missed tumor. The approach is minimally invasive and the nose heals quickly, especially with an intact mucosa on the contralateral sphenoid ostium and proper medialization of the middle turbinate at the end of the procedure. Our results, further, have been comparable to most endoscopic series, with a very low complication rate (Table 3).

**Table 3.** Mono-nostril vs. bi-nostril approach

	**Mono-Nostril**	**Bi-Nostril**
Working space	Crowded	Wider space
Dynamism	Less	More
Resection rates	Slight limitation invasive adenomas	Higher in large invasive tumors
Nasal healing	Faster	Slower
Crusting	Less/intact contra-lateral mucosa	More/Bilateral mucosal work

In conclusion, we have reviewed our experience with the mononostril endoscopic approach for pituitary tumors in 64 patients. We have shown comparable results to the binostril technique, mostly reported in endoscopic series, especially in secreting and non-invasive macroadenomas, as the endoscope allows inspection of the hidden corners and suprasellar region, allowing for a more nearly complete resection. The recurrence and complication rates were quite low, mostly limited to recurrent, or invasive adenomas. We feel that the mononostril approach is simple, less traumatic, and sufficient for pituitary adenoma surgery to achieve a good outcome.

Limitations to the mononostril approach may be a crowded narrow nasal cavity, a harder tumor with invasive appearance or significant suprasellar extension, and lesions other than pituitary adenomas. The mononostril surgery may then be simply turned into a binostril, wider and more dynamic approach to allow for better dissection of such larger, harder, and more extensive tumors.
